# The Ins and Outs of Cerebral Malaria Pathogenesis: Immunopathology, Extracellular Vesicles, Immunometabolism, and Trained Immunity

**DOI:** 10.3389/fimmu.2019.00830

**Published:** 2019-04-17

**Authors:** Frederic Sierro, Georges E. R. Grau

**Affiliations:** ^1^Vascular Immunology Unit, Department of Pathology, Faculty of Medicine and Health, School of Medical Sciences, The University of Sydney, Sydney, NSW, Australia; ^2^Human Health, Nuclear Science, Technology, and Landmark Infrastructure, Australian Nuclear Science and Technology Organisation, Sydney, NSW, Australia

**Keywords:** cerebral malaria (CM), immunopathology, extracellular vesicle (EV), Immunometabolism, trained immunity

## Abstract

Complications from malaria parasite infections still cost the lives of close to half a million people every year. The most severe is cerebral malaria (CM). Employing murine models of CM, autopsy results, *in vitro* experiments, neuroimaging and microscopic techniques, decades of research activity have investigated the development of CM immunopathology in the hope of identifying steps that could be therapeutically targeted. Yet important questions remain. This review summarizes recent findings, primarily mechanistic insights on the essential cellular and molecular players involved gained within the murine experimental cerebral malaria model. It also highlights recent developments in (a) cell-cell communication events mediated through extracellular vesicles (EVs), (b) mounting evidence for innate immune memory, leading to “trained“ increased or tolerised responses, and (c) modulation of immune cell function through metabolism, that could shed light on why some patients develop this life-threatening condition whilst many do not.

## Introduction

Among protozoan parasites of the genus *Plasmodium*, seven species are able to infect humans to cause malaria. The two major species are *Plasmodium vivax* and *Plasmodium falciparum*, with the former accounting for the most cases and the latter being responsible for the majority of deaths (1). Plasmodia are dual host parasites (mosquito and mammals) and within the mammalian host, broadly two hepatic stages and one blood stage are defined. Malaria induces a wide spectrum of symptoms and signs which differ between affected individuals, for example, between adults and children. In 1–2% of cases however (2), infection leads to severe malaria that exclusively develops during the blood stage of the malaria parasite cycle. Severe malaria can include the following disturbances, singly or in combination: electrolyte and metabolite imbalance, severe anemia, pulmonary oedema with respiratory distress, jaundice, and its most severe manifestation: cerebral malaria (CM). CM is characterized by, seizures, coma, and death. In endemic regions of Africa, CM mostly affects children under the age of five while in Southeast Asia, it is observed mostly in young adults. CM lethality still is in the range of 15–25% with the best available treatments (3). Over 25% of survivors from CM are afflicted by life-long sequelae including sensory and cognitive impairment (4), epilepsy and physical disability. Thus, despite incremental progress in more than a century since the discovery of the malaria parasite, a deep understanding of this disease and its deadliest complication, CM, is far from complete, and development of effective therapies remains a high priority.

A comprehensive appraisal of the histopathology associated with CM or experimental animal models of CM, as well as its changes following characterized interventions, constitute a valid experimental approach to identify targetable disease development stages, cellular or molecular players. This could eventually lead to the identification of adjunctive therapies. Investigating the histopathology and pathophysiology of HCM (human cerebral malaria) in patients has ethical and technical constraints since pathological analysis of brain and most other tissues besides blood are limited to post-mortem observations. Thus, models of experimental cerebral malaria (ECM), caused by infection of susceptible mice with *Plasmodium berghei* ANKA (PbA), have provided important clues. ECM and HCM have more than twenty documented shared pathological features (5, 6). In both humans and mice, early lesions involve binding of infected erythrocytes (IE) to the brain microcirculation (7), albeit this is a minor feature in ECM compared to HCM. More recently the presence of IE in ECM has been confirmed (8), and found to correspond with development of vascular leakage. A cascade of sequestration of various leukocyte subtypes, including monocytes, macrophages, NK cells, CD4^+^ and CD8^+^ T cells, then ensues. In ECM, CD8^+^ T cells are eventually responsible for endothelial damage and the subsequent breakdown of the blood-brain barrier (BBB). In both patients and mice, neurological signs progress from seizures, ataxia and paralysis to coma and eventual death, and both are associated with brain oedema and hemorrhages.

In this review, we summarize the consensus and controversies surrounding the sequence of cellular events leading to the development of vascular neuropathology in experimental CM (ECM) and their correlates in human CM (HCM). Numerous aspects of the similarities and differences between HCM and ECM have been reviewed (5, 6, 9–13), clearly stating the limitations as well as the strengths of mouse model. A consensus paper about these issues has been published (14). Other recent findings in HCM have been detailed and discussed (15). We also highlight recent investigations extending beyond the targeting of specific cell types (e.g., monocyte subsets) into potential modulators or effectors of CM such as extracellular vesicles, diet, immunometabolism and trained immunity.

## Sequence of Events Leading to CM Immunopathology

Both HCM and ECM are associated with a sequential and marked accumulation of various immune cell subtypes in the brain microcirculation but histological studies paint somewhat different pictures between them. In ECM, a number of studies have provided evidence for an early requirement for CD4^+^ and for a late accumulation of CD8^+^ T cells while for other accumulating cell types, there are still controversies as to direct pathogenic roles (5, 6, 16, 17). HCM is characterized by a disease spectrum in children that differs from what is observed in adults. Three disease categories have been defined in pediatric HCM (CM1, CM2, and CM3) (18), occurring in 15, 56, and 29% of clinically defined cases, respectively. CM1 patients only display IE sequestration, whereas patients with CM2 have IE sequestration associated with other intra- and perivascular pathology, including immune cell infiltrates. Patients with CM3 fulfill the complete World Health Organization clinical criteria for CM, including unarousable coma associated with infection, but died of non-malarial causes. In South-East Asian adult patients, mainly IE sequestration has been observed (19). Here we summarize the elucidated sequence of these cellular events and the proposed roles attributed to different cell types.

## Activation of Endothelial Cells and Sequestration of Infected Erythrocytes and Platelets

A hallmark in HCM post-mortem observations is the distension of cerebral capillaries and venules containing IE and platelets. This sequestration involves predominantly late stage IE and is considered an immune evasion mechanism, as it removes mature stages of the parasites form the circulation. It is accompanied by systemic endothelial activation with upregulation of markers of endothelial cell (EC) activation such as ICAM-1, E-Selectin, CD36, and von Willebrand Factor (vWF), that all can serve as adhesion molecules for IE. Platelets are now considered key contributors to CM by providing an alternate, indirect mechanism for IE cytoadhesion (20). This is achieved by forming bridges between IE and ECs at sites of low endothelial adhesion molecule expression, as shown *in vitro* (21, 22). These *in vitro* results have been confirmed by the demonstration of intravascular platelet accumulation in HCM, as shown in Malawian (3, 23) and South-East Asian (19) patients. Aside from this pathogenic role, platelets can exert a protective effect in malaria, as shown *in vitro* (24) and *in vivo* (25, 26). More recently, these *in vivo* results have been challenged, both *in vitro* using *P. falciparum* IE and *in vivo* using *P. chabaudi* and *P. berghei*-infected mice. Results indicate platelets do not kill blood-stage Plasmodium at physiologically relevant effector-to-target ratios. Furthermore, adoptive transfer of wild-type platelets to CD40-KO mice, which are resistant to ECM, partially restored ECM mortality and signs in CD40-KO recipients, indicating platelets are integral to the pathogenic process, and platelet CD40 is a key molecule (27), confirming 2002 data of Piguet et al. (28), *vide infra*. Sequestration of IE and platelets during ECM was originally disputed but further observations have also documented these as key pathogenic elements, as are the associated activation of ECs and upregulation of adhesion molecules.

This central and initiating EC-driven element of CM pathology is modulated by cytokines such as interferon-γ, TNF (previously called TNF-alpha) and lymphotoxin (previously called TNF-beta) (5). High levels of pro-inflammatory cytokines, in particular TNF, have been detected in the blood and the brain of patients who succumbed from CM as well as in models of ECM (29). Further supporting evidence has been provided for this scenario: *in vitro*, exposure to TNF induces upregulation of EC activation markers and IE pro-adhesion factors, and leads to increased IE binding on isolated human (30) or murine (31) brain microvascular cells.

## Immune Cell Accumulation

**Figure 1:**
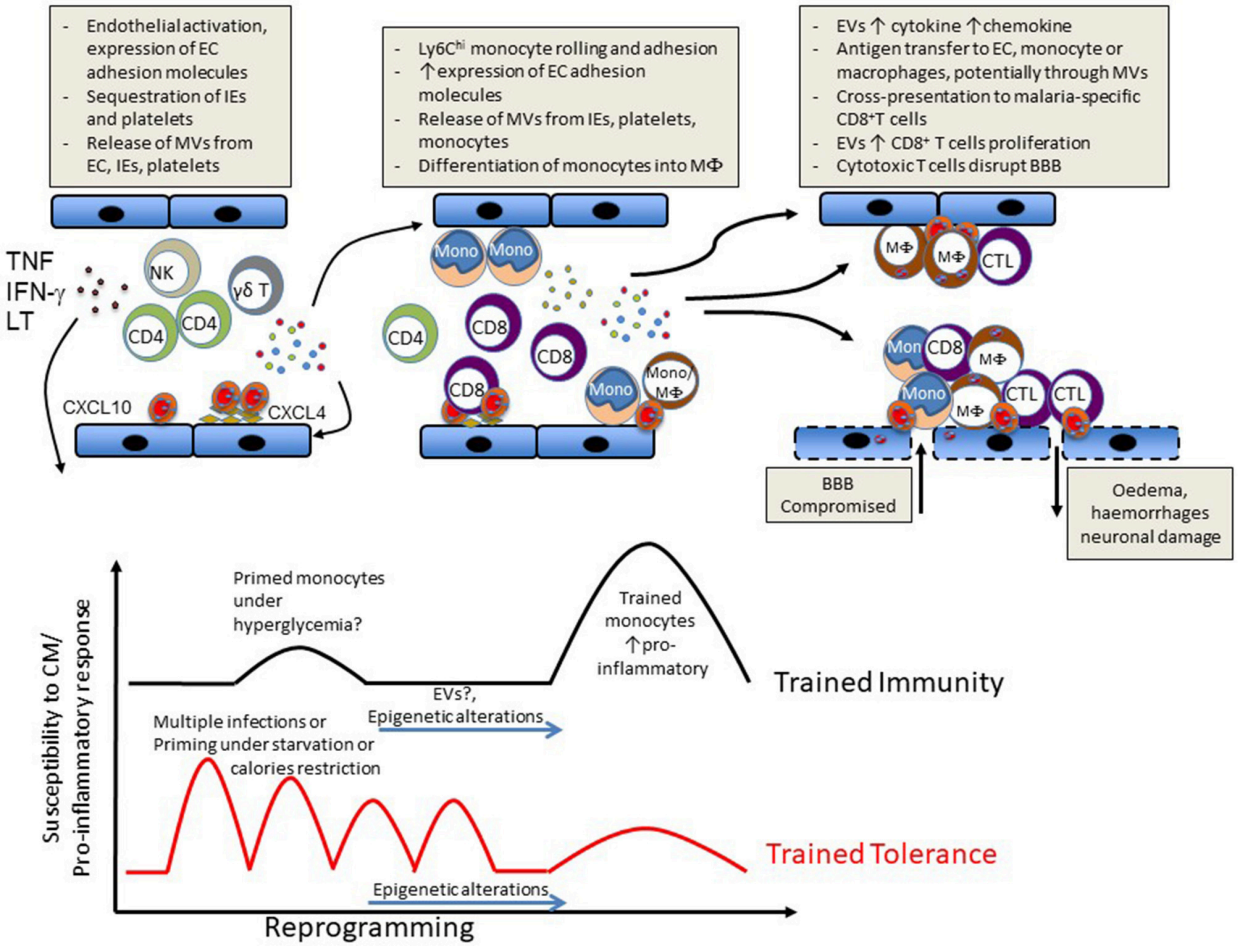
Schematic representation of events likely to lead to CM **(Upper)** or to influence susceptibility vs. disease tolerance to CM **(Lower)**.

Subsequent to the accumulation of IE and platelets, the intravascular accumulation of leucocytes is considered a key step in the development of ECM (32) and in some cases of HCM (3, 19, 23, 33). In ECM, this accumulation of host leucocytes in the brain microvasculature is correlated with the onset and severity of neurological signs and is thus considered to be largely responsible for neurological damage. While these immune subsets have been shown to mainly comprise monocytes, T cells and natural killer (NK) cells, their accumulation sequence and the relative pathological role of these and other cell subsets remain debated (Figure 1).

Numerous ECM studies suggest that accumulation of T cells (both CD8^+^and CD4^+^) cells in the brain of infected mice have major pathogenic roles. Supporting evidence has been obtained through anti-CD4 or anti-CD8 antibody depletion strategies (34, 35) just before or after the detection of neurological signs, or through the use of genetically deficient mice lacking functional CD8^+^+ T cells (32, 36, 37). In these models, infected animals failed to develop ECM, or showed a reduction in the severity of the syndrome. RAG2-KO mice that have been shown to be resistant to ECM can develop this pathology following adoptive transfer of CD8^+^ T cells isolated from PbA-infected ECM-susceptible WT mice. Furthermore, transfer of cells originating from perforin- or granzyme B-deficient mice does not result in ECM development, indicating an essential role for these cytotoxic molecules and supporting an effector function for CD8^+^ T cells. On the other hand, CD4^+^ T cell involvement in ECM pathogenesis appears to take on a helper role, as in the majority of studies and mouse strains used, their antibody-mediated depletion only prevents ECM development when performed before or early after infection (5, 34, 38, 39).

Evidence has documented a requirement for a concomitant presence of both T cells and IE for ECM development (40) as, in brains of mice infected with a parasite not causing CM, accumulation of CD8^+^ T cells is still present but no IE sequestration is observed. Conversely, experimental depletion or blockade of T cell accumulation in ECM models correlates with a diminution of IE accumulation (38, 41). While the proportion of sequestered T cells that are parasite antigen-specific is not known, parasite antigen-specific T cells do sequester within brain microvessels during ECM (42). Blockade of the adhesion molecule LFA-1 in a therapeutic setting (i.e., after onset of clinical signs) has been shown to be effective in blocking ECM and promoting survival (43, 44). A recent study confirms these earlier observations and provides a potential mechanism by showing that late, combined administration of antibodies specific for VLA-4 and LFA-1 decreases CD8^+^ T cell adhesion and numbers in cerebral blood vessels (39). Altogether, a current paradigm favors a mechanism involving high systemic levels of certain cytokines such as IFN-γ and TNF as well as binding of IE to brain vasculature. Both activate ECs which further increase binding of IE and allow recruitment and binding of T cells. These events participate in cross-presentation of malarial antigens accumulated on the luminal surfaces of cerebral blood vessels with parasite-specific CD8^+^ T cells previously primed in the periphery. These CD8^+^ T cells then cause local damage to the endothelium through cytotoxic mechanisms with ensuing BBB breakdown and neurological damage (30, 45).

While the view that T cells have a central role in ECM pathogenesis is undisputed, its translation to the human disease is less clear as there is little evidence for high numbers of CD8^+^ T cells in HCM. In ECM, this CD8^+^ T cell-centric model follows a neat sequence of events, but still presents some mechanistic gaps. Thus, several other cell types have been investigated and have been shown to be critically important. For example, local cross-presentation of parasite-derived antigens is unlikely to be performed by IE as they are devoid of MHC molecules. Several reports have suggested that activated ECs could perform this function after transfer of malarial antigens (30, 46–48). Others have repositioned myeloid cells in the spotlight, particularly intravascular sequestered monocytes that have long been reported in both HCM (3, 23, 33, 49, 50) and ECM (29). In ECM, we have shown, using intravital two-photon microscopy, that monocytes start accumulating in the brain several days prior to the onset of clinical signs (51). As the severity of the disease increases, slower rolling speed and increased adhesion of monocytes is directly observed in correlation with EC activation. Using high-dimensional flow cytometry we have calculated that at the onset of clinically-overt CM in mice, CD11b^+^ cells constitute ~80% of accumulating leukocytes in the brain vasculature at a time when T cells, B cells, neutrophils, DC, and NK cells remain numerically minor (52). Decreased ECM signs and pathology after global monocyte/macrophage depletion by clodronate liposomes (51) or after the abrogation of inflammatory monocyte recruitment by anti-CCR2 antibody (53) support a pathogenic role of these cells. The exact mechanisms by which inflammatory monocytes are involved in ECM pathogenesis still remain to be ascertained. Naturally, monocytes constitute a major source of cytokines and chemokines in CM pathogenesis and locally arrested monocytes could potentiate brain EC activation and recruitment of other leukocytes, notably CD8^+^ T cells. We have confirmed this by showing the interdependent recruitment of monocytic cells and CD8^+^ T cells to the central nervous system (51). Monocytes have the potential to differentiate into macrophage- or dendritic cell (DC)-like phenotypes. A recent study has reported that the majority of CD11b^+^ cells whose numbers peak at the onset of ECM symptoms also express CD11c, F4/80 and high levels of MHCII. These authors conclude that these are inflammatory monocytes that differentiate into splenic monocyte-derived DCs and are then subsequently recruited to the central nervous system (54). As opposed to resident macrophages and conventional DCs, a clear delineation between monocyte-derived DCs and monocyte-derived macrophages is still hotly debated, as extensively reviewed elsewhere (55). Recently, we have applied multiparametric high dimensional analysis using t-distributed stochastic neighbor embedding (tSNE) to further characterize the cerebral CD11b^+^ populations in ECM. This has allowed us to identify a population of non-resident Ly6C^lo^ cells with a monocyte/macrophage phenotype as the most prevalent population in the CM brain vasculature at peak disease. Importantly, we have further demonstrated that these monocytes derive from Ly6C^hi^ precursors and are pathogenic. Indeed, immune-modulatory particles (IMP)—previously shown to specifically target Ly6C^hi^ monocytes via induction of their sequestration in the spleen (56)—dramatically reduce CD11b^+^Ly6C^lo^ numbers in the brain and protect against cerebral and pulmonary lesions, with evidence of a down-modulation of immunopathology (52). Moreover, we have shown that when used in combination with established anti-malarial drugs, IMP treatment is highly effective (88% survival) even when given after neurological signs are present.

In addition to their protective role during malaria (57), γδ T cells have also been suggested as contributors to pathogenesis as they were found in patients (58), to be one of the predominant sources of cytokines and chemokines associated with severe malaria (50) Protection from ECM development following antibody-mediated depletion of γδ T cells supports this notion (59). However, γδ T cell depletion only prevents ECM development when performed before or early post infection, and such an effect is not observed in mice genetically deficient in γδ T cells.

Subsets of innate lymphoid cells (ILC) have been suggested to have distinct roles in CM. While expansion of ILC2 prevents ECM via a mechanism dependent on M2 macrophages and T regulatory cells (60), there is evidence that ILC1, through their cytotoxic NK subset, are involved in pathogenesis (61). However, once again, NK cell depletion prevents ECM, but only in a prophylactic setting.

Thus, while targeting many immune cell types prevents the development of ECM, it is only the direct targeting of CD8^+^T cells or of monocytes that has shown therapeutic effectiveness. The highly effective late stage success of the combination treatment with IMP and established anti-malarial drugs (52) highlights the novel potential of immunomodulatory targeting of innate immune cells in addition to CD8^+^ T cells in severe malaria, with a potential avenue for human translation where the role of CD8^+^ T cells is still unclear.

## Extracellular Vesicles: Both Immunomodulators and Effectors of Pathology

A hint of a role of membrane microvesicles (originally called microparticles) in immunopathology was adduced from experiments in which we attempted to understand the effector mechanisms of TNF in microvascular damage. Among these was the demonstration that TNF dose-dependently enhanced the release of microvesicles by endothelial cells (62). In parallel, microvesicles were found to be overproduced in the mouse model of CM. The majority of these microvesicles were from platelet origin, and seemed to be of pathogenic importance. Treatment of infected mice with an anti-CD40L mAb reduced microvesicle levels and thrombocytopenia, suggesting that CD40L is the main effector of malarial-induced thrombocytopenia. A role for platelet caspases *in vivo* was demonstrated by treatment of infected mice with the caspases inhibitor ZVAD-fmk, which reduced CM-associated mortality (28).

We subsequently found high microvesicle levels in the plasma of Malawian patients with CM. Remarkably, these high plasma levels were not seen in patients with severe malarial anemia, suggesting a relationship between microvesicle overproduction and the neuropathology (18). Since then, evidence has emerged that an important part of the immunopathological reaction is the release of extracellular vesicles, notably microvesicles (6) (Figure 1). Indeed, an effector role for microvesicles in immunopathogenesis is supported by the following evidence: (1) microvesicles can alter endothelial cell phenotype and function (63). (2) microvesicles are strong pro-inflammatory elements (64). (3) interfering with microvesicle binding to target cells reduces their activation (65), and (4) passive i.v. transfer of purified microvesicles exacerbates the disease in malarial-infected mice, and *in vitro* generated endothelial microvesicles even trigger CM-like lesions in naïve recipients (13).

We have found that ABCA1 deletion, which perturbs microvesicle release (66), fully protects mice against CM (67). Furthermore, blocking microvesicle production pharmacologically is beneficial for endothelial integrity (68, 69) and can prevent mortality due to ECM (70). Of note is that not only host-derived microvesicles appear important in CM: we have also shown that microvesicles released by malaria-infected erythrocytes *in vivo* are strongly pro-inflammatory in ECM, as evidenced by increased TNF production and CD40 expression on macrophages (64). This finding is not restricted to the experimental mouse model: plasma levels of erythrocytic microvesicles also are elevated in CM patients (71, 72).

Additionally, microvesicles have the potential to participate in antigen presentation, to express accessory molecules (48) and to amplify T cell proliferation, a property that we also had demonstrated in the context of TB (73). These results, together with those of Ramachandra et al. (74), suggest that microvesicles can be immunomodulatory elements, in addition to their effector roles in immunopathogenesis.

The involvement of microvesicles in CM remains incompletely understood because flow cytometry presents some limitations for their quantitation and characterization (75). Plasma microvesicles appear to carry discrete sets of miRNAs in relation to CM development (76). Malaria parasite themselves directly lead to the release of EV that contain small regulatory RNAs (77). Other aspects indicating a wider involvement of extracellular vesicles in malarial pathogenesis have been recently reviewed (78, 79).

## Immunometabolism and CM Development

The switch from oxidative phosphorylation to aerobic glycolysis and glutaminolysis was first described by Otto Warburg close to a century ago in the case of rapidly proliferating cancer cells (80). Since then it has become clear that reprogramming of transcriptional but also metabolic programs is a required intrinsic cellular adaptation to adjust tissue function in response to stresses. Injury and/or repair responses have been shown to drive metabolic changes to accommodate increased or specific demands. Proper organismal metabolic state adjustment is considered critical in maintaining disease tolerance while maladaptation is seen as contributing to pathology or long-term sequelae. These metabolic changes can be triggered by a plethora of signals such as certain cytokines or hypoxia. The mechanistic target of rapamycin complex 1 (mTORC1), a protein kinase complex expressed in most eukaryotic cells, is considered a critical signaling integrator that links these stimuli as well as nutrient sensing to the coordinated regulation of cellular metabolism (81, 82).

Following a seminal paper demonstrating that the co-stimulation of T cells with anti-CD28 increased glucose uptake and glycolysis (83), the same principles of metabolic reprogramming have been shown to be as critical in other immune cells in response to cytokines, antigens (84) or pathogen-associated molecular patterns (PAMPS) (85) and damage-associated molecular patterns (DAMPS).

In turn, the metabolic status at the cellular level affects and constrains cellular functional polarization, e.g., in key developmental steps or in pro- vs. anti-inflammatory polarization of immune cells. One of the mechanisms highlighted is the potential role of some metabolites as stabilizers for transcription factors such as in the case of succinate build up following OXPHOS disruption acting as an inflammatory molecular switch through stabilization of HIF1-α. This realization has resulted in a partial re-definition and extension of the field of immunometabolism from well-known regulatory roles of the immune system on local and systemic metabolism (tissue immunometabolism) to the additional modulatory role of cell intrinsic metabolic pathways on immune functions (cellular immunometabolism) [reviewed in (86)].

For example, it has been increasingly appreciated that T cell activation results in metabolic reprogramming from oxidative phosphorylation (OXPHOS) to glycolysis to meet the increased energetic and biosynthetic demands for T cell expansion and effector functions (87, 88). It was also conceived that promotion of effector cell differentiation, memory recall response or, on the contrary, regulatory phenotypes can be achieved by shifting their metabolism through changes in the availability of specific nutrients such as glucose or short chain fatty acids (SCFAs) (89, 90). In the case of monocytes/macrophages, the common view is that glycolysis supports inflammatory phenotypes or is favored after activation (91), while OXPHOS, presumably for sustained energy production, is a feature of resting monocytes/macrophages or macrophages with anti-inflammatory phenotypes. However, recent developments imply a more complex situation as glucose metabolism is still required in both anti-inflammatory and inflammatory macrophages (92, 93) and OXPHOS can also drive inflammasome activation in inflammatory macrophages (94).

Significant metabolic changes such as hypoglycaemia and lactic acidosis as well as perturbations in amino acid metabolism have long been observed in severe malaria in both ECM (95–97) and HCM (98–100). High brain concentrations of lactate, alanine, and glutamine are present in mice developing ECM but not in mice resistant to ECM (95, 101). We have demonstrated that elevated lactate was not uniform but occurred at the site of immunopathology, and that malaria parasites were not the dominant source of elevated lactate (102). These metabolic changes are consistent with, and have been mainly attributed to the occurrence of hypoxia/ischemia that follows obstruction of brain microvessels as in the case of ECM, both types of oedema, namely cytotoxic, and vasogenic oedema, have been demonstrated (97).

However, the recent developments in our understanding of immunometabolism mentioned above have called for a reassessment of hypotheses on mechanisms leading to CNS metabolite changes during CM development. It had been observed for some time that dietary restriction could prevent development of ECM, although only in a prophylactic setting (103, 104). Several recent studies have provided mechanisms on how manipulation of metabolism could explain this phenomenon. In independent studies, Gordon et al. (105) and Mejia et al. (106) demonstrated that caloric restriction or treatment with rapamycin, an inhibitor of mTOR, blocked the development of ECM. Remarkably, another study (107) showed that manipulating the glutamine pathway using the glutamine analog 6-diazo-5-oxo-L-norleucine (DON) rescues mice from ECM even when administered late in the infection. While the mechanisms proposed for these observations relate to blocks in activated T cell metabolism, few changes were observed in the numbers of T cells and other leucocytes recruited to the brain, and thus effects on other immune or mesenchymal cells remain a possibility. Indeed, a more recent report has shown that inhibition of glycolysis using the competitive glucose analog 2-deoxy glucose [2DG] was protective in ECM, not through effects on parasitaemia, the extent of anemia, the degree of cerebral oedema, or neuroinflammation but rather through modulation of haemostasis (108). These recent results emphasize the contribution of altered metabolic regulation to ECM and, more generally, to malarial pathogenesis in mice. The exact mechanisms behind these effects, the main cellular players involved, as well as well as their validation in HCM still requires investigation.

Because metabolic reprogramming can occur in response to changes in nutrient and oxygen availability, and in response to cytokines or other immune receptor stimulation, it presents the opportunity to therapeutically target these mechanisms at different levels, such as through dietary intervention. For example, pre-exposure to a high-fat diet reduced ECM via a mechanism that involved antioxidants (109). Another exciting prospect is the potential role of EVs in mediating such metabolic reprogramming. EVs could modulate metabolic pathways by virtue of transfer of miRNAs, mRNAs, proteins, and packaged metabolites to target cells. Such mechanisms have been described in response to other challenges such as retemplating of hepatic metabolic pathways through muscle-derived exosomes following intense exercise (110). As developed above, MVs and, to a lesser extent, exosomes are an essential component of CM development in both ECM and HCM but such link between the release of MVs in CM and immunometabolism regulation remains to be investigated.

## “Innate Immune Memory” and Trained Immunity in CM Development

“Trained immunity” is defined as the capacity of some innate immune cells such as monocytes/macrophages to display an enhanced or polarized immune response upon non-specific restimulation (111). Mechanistic studies have highlighted long-term epigenetic changes as being largely responsible for this phenomenon or the converse, the situation where a previous trigger leads to a decreased responsiveness to a subsequent immune challenge [reviewed in (112)]. These recent observations have cemented the long-proposed concept of “innate immune memory” (113, 114). The increased proinflammatory cell programs characterizing trained immunity or, on the contrary, “tolerised” innate immune phenotypes are associated with changes in cellular metabolism such as the metabolic shift from OXPHOS toward glycolysis occurring in *Candida albicans*-derived ß-glucan-induced trained immunity (111, 115).

An important recent discovery is the observation that metabolites such as succinate and fumarate can serve as modulators of epigenetic enzymes such as histone and DNA demethylases (116). Thus, the buildup of these TCA cycle metabolites or some derivatives such as aconitate (117) following OXPHOS disruption has been proposed to play a major role in innate immune memory. Such a link between metabolic reprogramming and trained immunity has the potential to provide novel insights into the pathogenesis of several diseases. Therefore, ongoing research is seeking to understand how innate immune memory, in some cases via immunometabolic modulation, could provide enhanced protection against reinfection, heterologous benefits against unrelated pathogens or on the contrary increase risks of post infection host-mediated pathology and increased susceptibility to chronic inflammatory diseases.

While beyond the scope of this manuscript, a large number of epidemiological studies have shown that outcomes of malarial infections are influenced by age and previous exposure. Furthermore, in specific resistant or susceptible ethnic groups where frequencies of single nucleotide polymorphisms (SNPs) in classical malaria-resistance genes cannot explain interethnic differences, epigenetic modifications or different chromatin landscapes were observed on specific host gene promoters or host genes that provide resistance to or susceptibility to malaria (118–121). In this context, there is also growing evidence that parasite infection can induce a state of trained innate immunity as exemplified by the hyperresponsiveness to TLR ligand stimulation of peripheral blood mononuclear cells (PBMCs) from patients with acute febrile disease *in vitro* (122). Additionally, higher numbers of IFN-γ producing PBMCs obtained following *P. falciparum* peptide stimulation in children suffering from malaria were correlated with significantly lower rates of malaria reinfection (123, 124). Approaches using controlled human malarial infections or vaccine trials have reported the induction of memory-like NK cells able to produce IFN-γ upon recall with malarial antigen or vaccine peptides. (125–127). A recent study has shown that PBMCs previously exposed to *P. falciparum* display an enhanced response to subsequent TLR2 stimulation and that this hyperresponsiveness correlated with increased methylation at specific proinflammatory and immunometabolic promoters. Importantly, these epigenetic modifications were also seen in Kenyan children infected with malaria (121). To our knowledge, there is no report of a direct link between trained immunity and CM. However, it is reasonable to hypothesize that such enhanced response training, although beneficial in fighting the parasite and assisting adaptive immunity, may increase the risk of severe disease upon reinfection. On the other hand, multiple episodes of malarial infection could instead induce tolerance to subsequent infection through modulation of this innate immune memory (Figure 1).

Much work remains to be done in order to integrate the interactions between immunologic signals, metabolic changes and epigenetic modifications with long-term changes in “innate immune memory” in response to malaria. Deciphering these mechanisms could have far reaching implications. It could help predict the risk of developing CM in individuals with previous (including *in utero*) malarial exposure. Modulation of trained immunity would also influence the clinical development of rationally designed malaria vaccines. Finally, this knowledge may help re-train innate cells during or before the disease to prevent severe complications. One could imagine that EVs may provide a way to achieve this by shuttling a designed cargo of miRNAS, epigenetic modifying enzymes or metabolites.

## Conclusion and Future Directions

The ECM model has produced a wealth of information on CM pathogenesis in mice with the aim to find an adjunctive therapy for HCM but its validity has been questioned. However, several investigators have provided a critical and evidence-based defense of this model (17, 128–131) and from knowledge gained from it, numerous laboratories have tested preclinical therapeutic interventions. Many have demonstrated efficacy at blocking the development of ECM but disappointingly, in a majority of cases, this was only found when administered before or early post infection and prior to the onset of clinical neurological signs. Therefore, only a few can justify, as therapies, a large-scale evaluation in HCM. A rare case of efficacy of treatment even when administered after the onset of clinical signs in the ECM model is the injection of IMP (52). The only other studies to date having demonstrated treatment efficacy after the onset of ECM have targeted CD8^+^ T cell binding to endothelial cells, and finally, immunometabolism. These successes have renewed hopes that the mouse model of ECM will continue to bring novel ideas/concepts which then will need to be confirmed or infirmed in HCM. For example, the characterization of retinal pathology in ECM (132) and (133), which was followed by the demonstration of its usefulness in HCM (134–137) (and many others) or MRI findings of brain alterations, originally described in 2005 for ECM (97), and followed by similar findings in HCM (138, 139).

In addition, it should be emphasized that the massive reduction in malaria burden achieved in the last two decades was mostly a result of prevention strategies rather than treatment. In this context, knowledge gained from ECM studies should not be seen as limited to the design of therapies but could also guide the expansion of our arsenal for HCM prevention. In particular, understanding changes in innate immune memory preconditioning and metabolic status in populations with high or low incidence of HCM could present opportunities for prevention through environmental or dietary interventions.

## Author Contributions

This review was co-written by FS and GG.

### Conflict of Interest Statement

The authors declare that the research was conducted in the absence of any commercial or financial relationships that could be construed as a potential conflict of interest.
